# What Predicts Visibility Management at Work? A Study of Gay, Lesbian, and Bisexual Flemish Government Employees

**DOI:** 10.5334/pb.443

**Published:** 2019-02-13

**Authors:** Alexis Dewaele, Mieke Van Houtte, Ann Buysse, Alona Lyubayeva, Michiel Trippas, Ann-Sophie Baeken

**Affiliations:** 1 Faculty of Psychology and Educational Sciences, Department of Experimental Clinical and Health Psychology, Ghent University, BE; 2 Faculty of Political and Social Sciences, Department of Sociology, Research team CuDOS, Ghent University, BE; 3 Diversity Policy Unit, Agency for Government Personnel, Flemish Government, BE

**Keywords:** work environment, visibility management, sexual orientation

## Abstract

Visibility management (VM) refers to the regulation of disclosure of one’s sexual orientation for the purposes of maintaining privacy as well as minimizing stigma, harm, or marginalization. Research on how lesbian women and gay men (LGs) manage the visibility of their sexual orientation in the workplace is scarce. In this study, we tested a model that investigates the relationships between VM on the one hand, and specific job characteristics, experiencing the work environment as more or less LG friendly, and personal homonegative experiences on the other. In a non-representative sample of 4,080 employees of the Flemish government, 6.3% identified as gay or lesbian. Within this LG subsample (*N* = 265) we found that specific job characteristics (having a managerial position, or having a tenured or non-tenured position) were not associated with VM. Knowing other LGs within the work environment who are open about their sexual orientation was associated with being more likely to apply open VM strategies, as was perceiving the atmosphere at work as permissive towards LGs. Having witnessed negative events towards LGs at work was associated with taking the characteristics of a social setting (e.g., public or private) into account when deciding to disclose one’s sexual orientation. Finally, participants who experienced homonegative events (such as unsolicited sexual innuendo or abusive language) felt less inhibited about disclosure. Potential theoretical as well as practical implications are discussed.

## Introduction

Being open about one’s sexual orientation as a gay man or lesbian woman (LG) can have negative consequences in the workplace (Ragins & Cornwell, 2001; Rumens & Broomfield, 2012). Professional environments—even contexts that LG employees define as LG friendly—may still involve forced choices between acceptance and visibility (Vincke, Dewaele, Vanden Berghe, & Cox, 2008; Williams, Giuffre, & Dellinger, 2009). LGs often report anticipating discrimination and hiding their sexual orientation at work as they fear negative consequences such as being bullied, missing out on career opportunities, or even forced resignation (Black, Makar, Sanders, & Taylor, 2003; Vincke et al., 2008). Increased attention on affirmative action and diversity policies at work (see e.g., Colgan, Creegan, McKearney, & Wright, 2007; Kalev, Dobbin, & Kelly, 2006; Ozeren, 2014) has shown that it is particularly relevant to explore dynamics that might explain the relationship between having a minority status as an employee, and specific behavior such as hiding a minority characteristic. However, few studies have investigated what predicts LGs to be open or closed about their sexual identity within the work environment. Studying these predictors is necessary since there does not always seems to be a straightforward relationship between anticipating discrimination on the one hand and being closed about one’s sexual orientation on the other (Kirby, 2006). Furthermore, this field of study has been hindered by narrow use of the dichotomous concept of coming out (i.e., that LGs are either open or closed about their sexual orientation). In this study, we preferred, instead, to use the concept of visibility management (VM). Visibility management refers to an ongoing process by which LGs make careful, planned decisions about what they will disclose and by which they continue to monitor the way in which they present their sexual orientation in different environments (Lasser & Tharinger, 2003). We tested a model that explores relationships between specific job characteristics, experiencing the work environment as more or less homonegative, and homonegative experiences on the one hand, and VM on the other. This study uniquely sheds light on how perceived work environments, as well as job characteristics, relate to behavior in LGs. Moreover, we explore whether the association between perceived LG-friendliness of the work environment and visibility management differs according to whether or not people have experienced homonegativity in the workplace.

### Managing Hidden Stigma and Developing a Sexual Identity

Identifying as lesbian or gay still implies the experience of stigma. Studies based on self-reported data show that LG workers report difficulties in coming out at work; experience rumors, innuendo and mockery; presume that talking openly about being LG might harm their professional careers; and even feel they may have missed an opportunity for promotion due to their sexual orientation (De Biolley, Aslan, Dewaele & Bonnet, 2007, Falcoz, 2008; Temmerman et al., 2016; Vincke et al., 2008; Wright, Colgan, Creegany, & McKearney, 2006). These experienced stressors might lead to self-censorship: LG workers refrain from being open about their sexual orientation simply because they can (Charpentier, 2004). After all, being lesbian or gay is not a visible personal characteristic.

Early literature already highlighted that the element of hidden stigma has an ability to generate anxiety in a person at risk of discreditation, as the individual fears being exposed as an impostor (Goffman, 1963). A cognitive-affective-behavioral process model outlines the psychological implications of concealing stigma: The ambiguity of social situations combined with the threat of potential discovery makes possessing a concealable stigma a difficult predicament for many individuals (Pachankis, 2007). One study showed that people with concealable stigmas (students who indicated that they were gay, that they were bulimic, or that their families earned less than $20,000 each year) reported lower self-esteem and more negative affect than both those whose stigmas were visible and those without stigmatizing characteristics (Frable, Platt, & Hoey, 1998). Smart and Wegner (1999) found that participants who conceal their stigmas become preoccupied with the control of disclosure of the stigmatized characteristic and therefore report more secrecy, suppression, and intrusive thoughts. According to the Disclosure Processes Model (Chaudoir & Fisher, 2010), antecedent goals representing approach and avoidance motivational systems moderate the effect of disclosure on numerous individual, dyadic, and social contextual outcomes. Long-term outcomes such as the impact on psychological health depend on whether one focuses on positive and negative goals of disclosure (e.g., attaining greater intimacy in relationships with others versus avoiding social rejection), the characteristics of the disclosure event (e.g., a confidant that reacts negatively), and mediating processes (e.g., a positive experience after disclosure that leads to increased social support from others). Chaudoir & Fisher (2010) also refer to feedback loops: The outcomes (positive or negative) of disclosure events influence whether one will maintain more open or closed VM strategies in the future (Chaudoir & Fisher, 2010). While the aforementioned theory refers to disclosure and VM as a continuous process, so is developing a sexual identity in LGs. As we will explain, both processes are interrelated.

Several early models that describe the development of a sexual identity in LGs refer to the association between a growing self-acceptance of one’s own sexual orientation as well as feeling the need to disclose this orientation to others (Cass, 1979, 1984; Coleman, 1990). Although the presumed linear pathways within these models have often been criticized (Horowitz & Newcomb, 2001), the notion that development of a sexual identity is characterized by some common characteristics between LG individuals (e.g., a process of self-labeling, growing self-acceptance, building up an LG social network, and increasing openness about sexual orientation) has been acknowledged by scholars (Elizur & Ziv, 2001; Rowen & Malcolm, 2002). The development of a sexual identity cannot be seen separately from an environment that is characterized (and perceived) as friendly versus hostile towards sexual minorities. The Minority Stress Model offers a conceptual framework for understanding how experiencing stigma, prejudice, and discrimination might cause mental health problems. It also explains how the expectation of rejection is associated with concealing one’s sexual orientation in order to cope with a potentially hostile environment (Meyer 2003, 2015). Being open about one’s sexual orientation might increase vulnerability. D’Augelli and Grossman (2001), for example, found that being more open was associated with more reported victimization in LGs. However, not every social context is comparable. and the work environment contains specific risks for individuals belonging to a stigmatized minority (e.g., being bullied on a day-to-day basis, missing out on a job opportunity). This highlights the need for a closer look to sexual minority VM strategies at work.

### Visibility Management Strategies at Work

Visibility management might function as a coping strategy to deal with potential discrimination whilst avoiding self-denial. There are various strategies LGs can adopt within the work environment: Chung (2001) differentiated between acting (i.e., engaging in a heterosexual relationship for the purpose of making people believe one is heterosexual), passing (i.e., fabricating information so that one may be perceived as heterosexual), covering (i.e., omitting or censoring information to avoid being identified as homosexual), being implicitly out (i.e., not labeling oneself as LG but being open and honest when it comes to information about one’s personal life), and being explicitly out (i.e., openly identifying oneself as LG). Disclosing at work and working for an organization perceived to be more LG-supportive has been found to correlate with positive outcomes such as a higher job satisfaction and lower job anxiety (Griffith & Hebl, 2002). Although this shows the importance of investigating VM strategies at work, there is little consistency in measuring these strategies. Several studies have measured disclosure as a one-off dichotomous event (e.g., age of first disclosure, years “out” since first disclosure; D’Augelli & Grossman, 2001; Pollitt, Muraco, Grossman, & Russell, 2017) and to being out *or* being closeted (De Brauwere, 2002, De Biolley et al., 2007). Others have looked at general disclosiveness, tapping into an individual’s style of disclosive behavior without referring to sexual orientation (Dew, Myers & Wightman, 2006) or assessing the number of people that know about the fact that a participant is LG (Griffith & Hebl, 2002; Legate, Ryan & Weinstein, 2012; Ragins, Singh & Cornwell, 2007; Rostosky & Riggle, 2002). Disclosure is also often narrowed down to “verbal communication that occurs between a discloser and a confidant” (Chaudoir and Fisher, 2010, p. 6). This ignores the fact that giving clues about one’s sexual orientation often involves the use of ambiguous language and a variety of symbols and nonverbal cues (Clair, Beatty & MacLean, 2005; Lasser & Wicker, 2008). Aforementioned assessments thus ignore the fact that VM is an ongoing process as described by theoretical models such as the Disclosure Processes Model (Chaudoir & Fisher, 2010). However, this limitation can be addressed by integrating a measure that refers to a continuous conceptualization of VM strategies (Lasser, Ryser, & Price, 2010). As such, VM refers to regulation of disclosure whilst doing away with the idea of maintaining one specific strategy all of the time (cf. Chung, 2001). It reflects an ongoing process by which LGs make careful, planned decisions about whether they will disclose their sexual orientation and by which they continue to monitor the presentation of their sexual orientation in different environments. It takes into account active behavior (e.g., showing to others that you are LG), inhibitive feelings (e.g., not feeling comfortable with disclosure) and also the role of social setting (e.g., thinking it is more suited to be open in a private compared to a public setting; Lasser & Tharinger, 2003). It thus provides a multidimensional perspective and transcends the dichotomous idea that individuals are out or not. However, this still leaves us with the question “what exactly determines these VM strategies at work?”.

### Determinants of Visibility Management Strategies at Work

Lower-socioeconomic status (SES) individuals tend to maintain a smaller bank of tangible interpersonal and intrapersonal resources to deal with stressful life events compared with higher-SES individuals (Gallo & Matthews, 2003). Therefore, it might be less easy for LGs in blue-collar jobs to be open about their sexual orientation, as less access to resources means higher risks. Also, as homonegative attitudes are associated with lower educational levels (Adolfsen, Keuzenkamp & Kuyper, 2006; Pickery & Noppe, 2007), work places with many blue-collar workers might be less LG-friendly and thus present a more complicated environment in which to be open (Ryan-Flood, 2004). However, some studies have shown that maintaining open VM strategies might be more difficult at higher levels of hierarchical systems. This might be explained by what is at stake for these employees: The higher their social status, the more they have to lose in terms of income and reputation (Trau & Härtel, 2004; Vincke et al., 2008). Also, jobs that require a significant level of authority might make openness in LGs more complicated (Claire et al., 2005). A qualitative study has shown that policemen who have disclosed their sexuality to colleagues still take precautions to manage the risk of them not being perceived as ‘hard enough’ to do the job (Rumens & Broomfield, 2012). This perception is not necessarily unwarranted, as, for example, students have been found to perceive a gay teacher as significantly less credible than a straight teacher (Russ, Simonds, & Hunt, 2002). Maintaining open VM strategies might thus threaten the ability of LG employees to occupy an authoritative status. Job characteristics such as hierarchies or the risks involved in occupying a specific position could influence VM strategies.

The perception LGs have of their work environment seems related to VM strategies, but, again, not necessarily in a straightforward way. For example, one study has shown that when LG students search for professional positions they often expect discrimination, although many will not let that fear force them back into the closet. Some students fear discrimination and hide their sexual orientation and, for example, choose not to list leadership activities in LG groups on their résumés, but many state that they will be out at work and risk discrimination in the long run because being true to themselves is worth the risk (Kirby, 2006). Nevertheless, in environments that offer high levels of support to autonomy (i.e., where people feel accepted for who they are and are free to act and express themselves) people feel less pressured to appear, behave, or perform in a certain way. For LG individuals this facilitates disclosure (Legate et al., 2012). LG workers are more likely to be open about their sexual orientation when their organizations have written, documented nondiscrimination policies that actively show support for LG activities, and offer diversity training that specifically includes LG issues (Griffith & Hebl, 2002; Rostosky & Riggle, 2002; Waldo, 1999). In fact, organizational support for LG employees also appears to be more fundamental to fostering an environment where it is okay to be open about one’s sexual orientation than support from supervisors and coworkers (Huffman, Watrous-Rodriguez & King, 2008).

Finally, most studies show that individuals who report past experience with discrimination on the grounds of sexual orientation are more likely to maintain more closed VM strategies (e.g., Frank, 2006; Vincke et al., 2008; Trau & Härtel, 2004). Negative experiences in the past generally contribute to being more closed in the future since a person is more likely to reveal their status if he or she believes that this will lead to positive outcomes (Clair et al., 2005). As internalized homonegativity (self-directed prejudice in LGs that originates from longstanding experiences with prejudice and discrimination in society, see Herek, 2009) has been systematically found to be related to more closed VM strategies (Dewaele, Van Houtte, & Vincke, 2014; Dew et al., 2006; Rostosky & Riggle, 2002), experiences of prejudice and discrimination might have a different impact when they are perceived as exceptional as opposed to common. For individuals who have often experienced prejudice and discrimination at work, current LG-friendly experiences might still not be enough to make them want to take the risk of coming out (Ferfolja, 2009).

### Hypotheses

**Figure 1:**
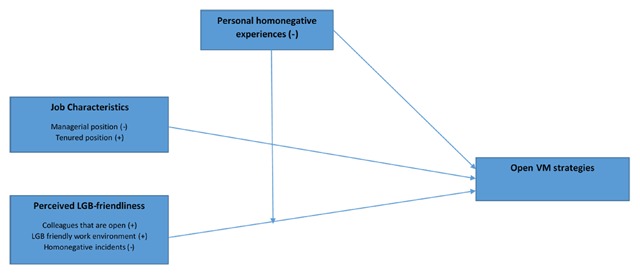
Model predicting open visibility management strategies (‘+’ en ‘–’ refers to a presumed positive/negative association with open VM strategies, respectively.

Although VM strategies tap into active behavior, inhibitive feelings, and the role of social settings (Lasser & Tharinger, 2003), lack of previous research makes it difficult to formulate specific hypotheses that include these different VM sub-dimensions. We will include the latter, however, for exploratory purposes. Our review of the literature suggests that VM might be related to the educational level of individuals occupying a particular job (Gallo & Matthews, 2003), as well as with the level of authority that is required for a specific job position (Adolfsen et al., 2006; Pickery & Noppe, 2007; Ryan-Flood, 2004). Furthermore, the Minority Stress Model predicts that the way in which LGs perceive their environment (i.e., homonegative or not) as well as whether they experience homonegative events at work, will be associated with VM strategies. Closed VM strategies will be maintained to avoid the negative consequences of stigma and to protect themselves from harm (Meyer, 2003, 2015). Finally, Chaudoir and Fisher’s (2010) Disclosure Processes Model states that the outcomes of VM strategies may shape individuals’ overall disclosure trajectories (i.e., feedback loops). When individual disclosure events create positive outcomes, they may serve to increase open VM strategies for workers at that organization. When individual disclosure events create negative outcomes, they may serve to increase future closed VM strategies. This translates into the following hypotheses (see Figure 1):

1. Job characteristics: We predicted that employees in managerial positions would have more to lose (both in terms of position and in terms of authority) than those who are not. Therefore we hypothesized that the former would maintain more closeted VM strategies than employees without a managerial position (H1.1). On the flip side to this, employees who have a tenured position have less to lose and therefore feel more secure than those who have not. We therefore hypothesized that the former would maintain more open VM strategies (H1.2).
2. Perceived LG-friendliness of the work environment: When LG employees have colleagues who are open about their sexual orientation this will act as an incentive to adopt open VM strategies (H2.1). We predicted that perceiving a work environment as LG-friendly, or witnessing homonegative incidents at work, would go together with more open (H2.2) and more closed (H2.3) VM strategies, respectively.
3. Personal homonegative experiences at work suffered by LG employees would be related to more closed VM strategies (H3.1). In line with the idea of feedback loops in the Disclosure Processes Model, previous homonegative experiences might also moderate the relationship between perceived LG friendliness of the work environment and VM: For those with negative experiences in the past, perceived LG friendliness might not relate to more openness. For those without negative experiences, we predicted that perceived LG-friendliness would be related to more openness (H3.2).

## Method

The data gathered for this study were commissioned by the Diversity Policy Unit for Emancipation within the Flemish Government. In 2004 the Flemish Government decided to promote and support equal opportunities and diversity policies within the Flemish administration. This translated into a Strategic Equal Opportunities and Diversity Plan that, amongst other goals, aimed to analyze the position of LG employees within the Flemish administration (Vlaamse Regering, 2015). The Flemish administration has 40,325 employees.

### Participants and recruitment

The software package ‘Opinio’ was used to develop and conduct the survey (www.objectplanet.com/opinio). It was required that responses were kept anonymous. Therefore an option offered by the software package to make tracking of invitees (whether they had responded to the survey or not) impossible was selected. On top of this, the IP check sometimes used to prevent multiple responses was disabled to improve anonymity. However, multiple responses were avoided as only one survey could be taken per laptop/desktop. All necessary technical and organizational measures to ensure safe processing of research data were applied in order to conform to the guidelines of the ethics committee of the Faculty of Psychology and Educational Sciences at Ghent University. The period of data gathering started on November 6^th^ and continued up until November 30^th^ 2014. To stimulate participation, participants were offered the chance to win cinema tickets if they were willing to enter their e-mail address at the end of the survey. Furthermore, several methods were used to recruit participants: Calls for participation were published on the home webpage of the Diversity Policy Unit, and on the general intranet message board as well as on specific billboards of several departments within the Flemish administration. A poster and flyer campaign was also launched, with building managers made responsible for spreading posters and flyers in the buildings of the Flemish administration in all Flemish provinces as well as in Brussels (the Belgian capital). A promotion team, set up from within the Diversity Policy Unit, dispersed flyers on several occasions. Diversity and communications officers within all departments and agencies got an invitation by e-mail to spread the call for participation and to motivate potential participants. They were encouraged to take action at a meeting halfway through November. Finally, members of the Rainbow Network for lesbian, gay, bisexual, and transgender employees within the Flemish administration were contacted to help distribute posters and flyers. Our sample includes 4,239 participants (i.e., 10.5% of all employees working within the Flemish administration). To the question “how would you describe your sexual orientation”, 88.2% responded “heterosexual”, 6.3% responded “lesbian/gay”, 1.8% responded “bisexual”, 0.5% responded “I do not know”, and 3.2% responded “I do not want to answer this question”. Research shows that sexual identity processes as well as VM strategies might be significantly different between gay/lesbian and bisexual individuals, with the latter reporting higher levels of identity confusion and lower levels of self-disclosure and community connection (Balsam & Mohr, 2007). Since we could not control for relationship status (whether one is in a romantic relationship with a man or a women), a characteristic that could be associated with VM strategies, we opted to exclude bisexual participants from the analysis. Therefore, this study was conducted on the subsample of participants who identified as lesbian or gay (*n* = 265). Our LG subsample (see Table 1) was made up of 45.6% women and 54.4% men. Since the subgroup of those who did not identify as either a man or woman, or who responded ‘I do not know’ or ‘other’, was too small to analyze, their scores were treated as missing values (2.3% of the LG subsample). The mean age of the participants was 38.93 years (*SD* = 9.27). With regard to educational level, we found that 2.6% had earned a degree of lower secondary education, 14.7% had earned a degree of higher secondary education, 40.8% had earned a bachelor’s degree, and 41.9% had earned a master’s degree. When we compared participants from the LG subsample with participants from the non-LG subsample (see Table 1), LG participants were more likely to be male (*χ^2^* = 55.61, *p* < .001), younger (*t* = 4.02, *p* < .001), and more highly educated (*χ^2^* = 14.5, *p* < .01) compared to participants from the non-LG subsample.

**Table 1:** Sample Characteristics: Absolute (N) and Relative Frequencies (%), Mean or Median, Standard Deviation and Results of Tests of Difference Comparing LG Sample with Non-LG sample.

	Total (*N* = 4,080)		LG sample (*N* = 265)		Non-LG sample (*N* = 3,815)		Test of difference

	*M/Mdn/%*	*SD*	*M/Mdn/%*	*SD*	*M/Mdn/%*	*SD*	

Sex							*χ^2^* = 55.61***
Female	66.7%		45.6%		68.1%		
Male	33.3%		54.4%		31.9%		
Age	41.17	10.38	38.93	9.27	41.33	10.44	t(311.50) = 4.02***
Education							
Lower sec.	4.1%		2.6%		4.2%		*χ^2^* = 14.5**
Higher sec.	23.3%		14.7%		23.9%		
Bachelor	37.1%		40.8%		36.8%		
Master	35.6%		41.9%		35.2%		
Managerial position							*χ^2^* = .13
Yes	14%		14.7%		13.9%		
No	86%		85.3%		86.1%		
Tenured position							*χ^2^* = 7.73**
Yes	37.1%		45.1%		36.5%		
No	62.9%		54.9%		63.5%		
Having LG colleagues							*χ^2^* = 34.89***
Yes	80.7%		94.6%		79.9%		
No	19.3%		5.4%		20.3%		
LG permissive atmosphere	4.34	0.79	4.26	0.82	4.34	0.78	t(4065) = 1.69
Negative events	1.28	1.27	2.12	1.59	1.22	1.22	t(277.29) = –8.88***
Homonegative experiences			0.70	1.03	N.a.		
VM Active			14.99	3.41	N.a.		
VM Inhibitive			14.50	3.73			
VM Setting			7.00	2.28			

* *p* < .05, ** *p* < .01, *** *p* < .001.

### Measures

#### Dependent variable

An abbreviated version of the LG *Visibility Management Scale* (VMS) (Lasser et al., 2010) was used, which contains three subscales: Active Behavioral, Inhibitive Behavioral, and Setting. The Active Behavioral subscale (six items) concerns behaviors used to make one’s sexual orientation visible. For example, “It is important to let others know about my sexual orientation.” The Inhibitive Behavioral subscale (five items) refers to feelings associated with disclosure of one’s sexual orientation. For example, “I feel comfortable talking about my sexual orientation.” Finally, the Setting subscale (three items) taps into the role that social setting plays in someone’s VM decisions. “Some settings are more appropriate than others for disclosing my sexual orientation” is a statement that is representative of the items for this last subscale. Each VM item was assessed using 5-point scales (0 = *strongly disagree* to 4 = *strongly agree*). Lasser and colleagues (2010) have previously found evidence for construct validity, item-level discrimination, and subscale reliability. Higher scores on the VM subscales indicate more expansive disclosure behaviors (Active Behavioral), less negative feelings towards disclosure (Inhibitive Behavioral), and that an individual takes the social setting less into account (Setting). In this study, the internal reliability ratings (Cronbach’s Alpha) for the VMS subscales were .59, .74, and .58, respectively.

#### Determinants

We distinguish three clusters of determinants: Job characteristics, perceived LG-friendliness of the work environment, and homonegative experiences (cfr. Pelssers et al., 2008, see Appendix for a description of items). *Job characteristics* were assessed with two dichotomous items. Firstly, participants could tick one of two boxes to answer whether they had a managerial position or not. Secondly, they could tick one of two boxes to answer whether they did or did not have a tenured position. *Perceived LG friendliness of the work environment* was assessed with items reflecting three different aspects: (1) Having colleagues that are open about their homosexual orientation (one dichotomous item, based on a *yes* or *no* answer), (2) perceiving a permissive work atmosphere towards LGs (rated on a 5-point scale, 1 = *strongly disagree* and 5 = *strongly agree*), and (3) having witnessed homonegative incidents (seven dichotomous items, based on *yes* or *no* answers, that were added into one summed score). Finally, five items were used to assess *homonegative experiences* at work. We asked participants if they had ever, while working for the Flemish government, experienced different types of homonegative experiences (five dichotomous items, based on yes or no answers, that were added into one summed score).

### Data Analysis

Spearman’s intercorrelations between all demographic, independent, and dependent variables were explored (see Table 2). To test our hypotheses a multiple regression analysis was conducted (see Table 3). In the tested model, we controlled for the effect of specific demographic variables since gender (Dewaele, Cox, Van Houtte, & Vincke, 2013), age (Elizur & Ziv, 2001; Weststrate & McLean, 2010) and educational level (Gallo & Matthews, 2003; Ryan-Flood, 2004) are all potentially associated with sexual identity development processes and/or the use of VM strategies. We tested the direct effects of these demographic variables as well as of job characteristics, perceived LG-friendliness of the work environment, and personal homonegative experiences on the dependent variable (VM). To avoid problematic effects of the skewed distribution of the variable “personal homonegative experiences at work” (skewness = 1.62, *SE* = .13; kurtosis = 2.23, *SE* = .26), we converted the scores of the variable to logarithmic scales. In a second model we tested H3.2 (previous homonegative experiences will moderate the relationship between perceived LG friendliness of the work environment and VM). Therefore we included interaction terms combining the three variables that measured perceived LG-friendliness of the work environment and the variable that assessed previous homonegative experiences (three interaction terms in total).

**Table 2:** Intercorrelations Between all Demographic, Independent, and Dependent Variables.

		1		2		3		4		5		6		7	8		9		10		11		12

1.	VM Active	1.000																					
2.	VM Inhibitive	.687	**	1.000																			
3.	VM Setting	.540	**	.656	**	1.000																	
4.	Sex	–.019		–.108		–.095		1.000															
5.	Age	–.045		.102		.033		.049		1.000													
6.	Education	–.023		–.021		–.005		.150	*	–.136	*	1.000											
7.	Position	.036		.027		.042		.103		.132	*	.102		1.000									
8.	Tenured	–.071		.030		.029		.030		.340	**	–.026		.068	1.000								
9.	Open	.194	**	.214	**	.136	*	–.009		–.042		.037		.050	–.011		1.000						
10.	Atmosphere	.155	**	.326	**	.207	**	.049		–.017		.046		–.001	–.212	*	.171	**	1.000				
11.	Witnessed	.142	*	–.032		–.083		–.007		.093		–.200	**	.011	–.015		.058		–.150	*	1.000		
12.	Experiences	.164	**	.058		.018		–.022		.112		–.181	**	–.030	–.002		.023		–.144	*	.706	**	1.000

*Note: Position* having a managerial position (versus not having one), *Tenured* having a tenured position (versus not having one), *Open* having colleagues that are open about their sexual orientation (versus not having LG colleagues), *Atmosphere* perceiving the atmosphere at work as permissive towards LGs, *Witnessed* having witnessed negative events towards LGs at work, *Experiences* personal homonegative experiences. * *p* < .05, ** *p* < .01.

**Table 3:** Linear Regression Analysis Predicting Visibility Management Strategies (VMS) from Job Characteristics, Perceived LG Friendliness of the Work Environment, and Personal Homonegative Experiences.

Predictor	*β*											

	Active				Inhibitive				Setting			

	Model 1		Model 2		Model 1		Model 2		Model 1		Model 2	

Sex	.02		.02		–.08		–.09		–.06		–.07	
Age	–.07		–.07		.03		.03		.01		–.01	
Education	.01		.01		.03		.02		.01		.01	
Position	.02		.03		–.02		–.02		.03		.03	
Tenured	.06		.05		.12		.12		.09		.10	
Open	.24	**	.20	*	.26	***	.22	**	.15	*	.16	*
Atmosphere	.14	*	.17		.34	***	.40	***	.21	**	.31	**
Witnessed	.10		.05		–.14		–.12		–.20	*	–.11	
Experiences (EXP)	.14		–.12		.21	*	.04		.15		.35	
EXP*Open			.23				.19				–.15	
EXP*Atmosphere			–.02				–.08				–.15	
EXP*Witnessed			.11				–.05				–.19	
Total *R*^2^	.12	**	.13		.23	***	.24		.11	**	.13	
*N*	265											

*Note: Position* having a managerial position (versus not having one), *Tenured* having a tenured position (versus not having one), *Open* having colleagues that are open about their sexual orientation (versus not having LG colleagues), *Atmosphere* perceiving the atmosphere at work as permissive towards LGs, *Witnessed* having witnessed negative events towards LGs at work, *Experiences* personal homonegative experiences. Model 1 includes demographic variables as well as variables that refer to job characteristics, perceived LG-friendliness of the work environment, and personal homonegative experiences. Model 2 includes all aforementioned variables as well as three interaction terms. * *p* < .05, ** *p* < .01, *** *p* < .001.

## Results

The bivariate analysis (see Table 2) revealed significant correlations between the determinants and scores on the different VM subscales (VM Active Behavioral, VM Inhibitive Behavioral, and VM Setting). Scores on the VM Active Behavioral subscale were positively associated with scores on the VM Inhibitive Behavioral subscale (*r* = 0.69, *p* < .01), scores on the VM Setting subscale (*r* = 0.54, *p* < .01), having colleagues who are open about their sexual orientation (*r* = 0.19, *p* < .01), perceiving the atmosphere at work as permissive towards LGs (*r* = 0.16, *p* < .01), having witnessed negative events towards LGs at work (*r* = 0.14, *p* < .05), and with personal homonegative experiences at work (*r* = 0.16, *p* < .01). Scores on the VM Inhibitive Behavioral subscale were positively associated with scores on the VM Setting subscale (*r* = 0.66, *p* < .01), having colleagues who are open about their sexual orientation (*r* = 0.21, *p* < .01), and with perceiving the atmosphere at work as permissive towards LGs (*r* = 0.33, *p* < .01). Finally, scores on the VM Setting subscale were associated with having colleagues who are open about their sexual orientation (*r* = 0.14, *p* < .05) and with perceiving the atmosphere at work as permissive towards LGs (*r* = 0.21, *p* < .01).

Multivariate analysis (see Table 3) showed that, after controlling for demographic variables (sex, age, and education), *job characteristics* were not associated with particular forms of VM.

Having a managerial or tenured position was not associated with scores on the VM subscales. This disproves hypotheses H1.1 and H1.2. Our hypotheses related to *perceived LG-friendliness of the work environment* were largely supported. We found support for H2.1: When LG employees have colleagues who are open about their sexual orientation, they report higher scores on the Active Behavioral, Inhibitive Behavioral, and the Setting subscales (β = 0.24, *p* < .01; β = 0.26, *p* < .001; and β = 0.15, *p* < .05 respectively). We also found support for H2.2: Perceiving the atmosphere at work as permissive towards LGs was associated with higher scores on the Active Behavioral, Inhibitive Behavioral, and the Setting subscales (β = 0.14, *p* < .05; β = 0.34, *p* < .001; and β = 0.21, *p* < .01 respectively). We found partial support for H2.3: Witnessing homonegative incidents at work was associated with lower scores on the VM Setting subscale (β = –0.20, *p* < .05) but not with scores on the Active Behavioral (β = 0.10, *p* > .05) and Inhibitive Behavioral (β = –0.14, *p* > .05) subscales. Furthermore, reporting a higher number of *homonegative experiences* was associated with higher scores on the Inhibitive Behavioral subscale (β = 0.21, *p* < .05) but not with scores on the Active Behavioral (β = 0.14, *p* > .05) and Setting (β = 0.15, *p* > .05) subscales. This contradicts H3.1. Finally, none of the interaction terms that were added to the second model were significant. We thus found no support for H3.2, that negative experiences moderate the relationship between perceived LG-friendliness of the work environment and VM strategies.

## Discussion

Few studies have used data from within specific work environments to analyze the VM strategies adopted by LGs at work. This is especially relevant as several studies have pointed out that difficulties can arise when sexual minority individuals choose to come out within a work environment. Negative consequences such as harassment, job loss, and direct and indirect discrimination have been reported frequently (Ragins & Cornwell, 2001; Rumens & Broomfield, 2012; Vincke et al., 2008). Yet little research has shed light on what exactly determines VM strategies at work. Moreover, no studies that we know of have explored how job characteristics and perceived work environment might contribute to VM strategies in LGs whilst measuring it as a continuous process rather than as a reductionist dichotomy (i.e., LGs are out or closeted). The way we measured VM (differentiating between disclosing behaviors, positive feelings associated with disclosure, and the role of social setting) has proven to be useful to test and better understand associations between job characteristics, perceived LG-friendliness of the work environment and homonegative experiences on the one hand, and VM strategies in LGs on the other.

In this study, we did not find job characteristics (having a managerial or a tenured position) to be associated with VM strategies. A self-determination theory framework (Deci & Ryan, 2008) would predict that for LGs who work in an environment that supports autonomy (which could be assumed to be the case for those who have a tenured versus a non-tenured position), being open would be easier. On the other hand, the need for maintaining authority, as is the case in a managerial position, might complicate coming out. The fact that these job characteristics did not associate with VM in our study might be explained by the fact that for individuals who feel safe in their position in the labor market, being open or not depends on factors other than job characteristics. Research has shown that working for the Belgian government provides a relatively safe environment for minorities, as wages are predetermined and, consequently, the risk of wage discrimination is limited (Vincke et al., 2008). Within this safe climate, other factors that we did not assess, such as sexual identity salience (e.g., how important is an LG identity for a person?) and career aspirations (e.g., how important is becoming a manager for me?) might play a more important role (see e.g., Ragins, 2004).

In our larger sample of both LGs and heterosexuals, 81% reported to have LG colleagues, and people tended to perceive their work environment as (rather) LG permissive (see Table 1). This all shows that this specific work environment is perceived as quite LG-friendly, at least by the people who participated in the survey. Nevertheless, we found that even within this permissive work environment, perceiving the atmosphere at work as permissive towards LGs and having LG colleagues was associated with more disclosing behaviors, more positive feelings associated with disclosure, and attaching lesser importance to the role of social setting for disclosure. In contrast, having witnessed negative events towards LGs at work was associated with perceiving the role of social setting as more important for disclosure. The minority stress model explains how minority stressors such as the experience of discrimination and prejudice relate to an individual concealing their sexual orientation. Maintaining closed VM strategies can therefore be seen as a coping strategy adopted by LGs to minimize the negative consequences of stigma (Meyer, 2003). On the other hand, as being open about one’s sexual orientation is also related to lower expectations of being rejected (Lewis, Derlega, Griffin, & Krowinski, 2003), the former might also lead to reduced sensitivity and less awareness of homonegativity. Finally, the fact that participants who have LG colleagues at work report being more open about their sexual orientation, shows the importance of role models within a work environment (Carrington & Skelton, 2003; Riggle, Whitman, Olson, Rostosky & Strong, 2008).

Unexpectedly, our study found that having repeatedly experienced homonegative events was associated with more positive feelings about disclosing one’s sexual orientation. At first glance, it seems to make no sense that LGs would feel more confident as a result of these negative experiences. Another study also showed that perceived past experience of sexual orientation discrimination was related to greater disclosure, however. Resilience in the face of perceived past discrimination can be understood within the context of the idea that disclosure is driven not only by fear, but also by the need to develop an authentic sense of self in the workplace (Ragins et al., 2007). More likely, though, is that the relationship might be the other way around, and that one has to be open about one’s sexual orientation in order to be rejected by individuals from the majority group (Dewaele et al., 2014). Consequently, those participants who have very strong feelings about the importance of being open about one’s sexual orientation, might also be the ones who elicit the most negative reactions from colleagues at work.

Finally, no support was found for the hypothesis that homonegative experiences moderate the relationship between perceived LG-friendliness of the work environment and VM strategies. This contradicts the idea of feedback loops outlined in the Disclosure Processes Model: That outcomes of VM strategies may shape the overall disclosure trajectory. When disclosure is followed by positive outcomes, they may serve to increase open VM strategies and vice versa (Chaudoir & Fisher’s, 2010). Maybe this shows, as we have already previously argued, that maintaining a sense of authenticity is more important for many LG employees compared to fearing the negative outcomes of disclosure. However, we should be careful in completely discarding the idea of feedback loops as certain elements that have been identified as important in the Disclosure Processes Model (Chaudoir & Fisher, 2010) remain unexplored in this study. For example, we did not measure whether participants focus on positive versus negative goals of disclosure (e.g., attaining greater intimacy in relationships with others versus avoiding social rejection).

This study had other limitations and strengths that should be taken into account. Firstly, the Flemish administration is not representative of all work environments. On the contrary, Belgium is ranked in the top five most LG friendly countries in Europe (Roelandt, Dewaele, Buysse, & Van Houtte, 2016). Within Belgium, the Flemish administration is an employer that actively tries to protect employees with a minority background whilst striving for a diverse workforce (Scheepers, 2008). Due to a lack of sampling frame, it is impossible to assess how representative our LG subsample is for all LGs working within the Flemish administration. Although we should be careful not to generalize these findings, they still shed light on a specific work environment occupied by government employees and illustrate how LGs manage their visibility within this specific context. Secondly, our data is limited because of its cross-sectional nature: Associations cannot explain causality. For example, an LG-permissive atmosphere can contribute to more openness in LGs, but it might also be true that more openness from individuals contributes to an LG-permissive atmosphere. Finally, low levels of Cronbach’s Alpha for some VM dimensions (Active Behavioral and Setting) suggest that the VM questionnaire could be further improved.

To summarize, this study shows that even within an LG-friendly country and work environment perceived homonegativity is still associated with more closed VM strategies. Experiencing homonegative events, was associated with more openness in LGs, however. This probably reflects the fact that openness about one’s sexual orientation makes one more visible as an LG, and therefore increases the odds of being the target of homonegativity. The results also seem to support the importance of role models (colleagues who are open about their sexual orientation). New studies could extend these insights by including objective indicators of LG-friendliness at work, testing models that predict VM in diverse work settings (LG-friendly as well as more homonegative environments), including more blue collar workers, and gathering longitudinal data to explore causal mechanisms.

## Additional File

The additional file for this article can be found as follows:

10.5334/pb.443.s1 Appendix. Items that assess different clusters of determinants.
