# Supplementary material for: What Predicts Visibility Management at Work? A Study of Gay, Lesbian, and Bisexual Flemish Government Employees
Source: Psychol Belg. 2019 Feb 13;59(1):78–95. doi: 10.5334/pb.443 (PMC6625549; doi:10.5334/pb.443)
Supplement: Appendix. — Items that assess different clusters of determinants. [file pb-59-1-443-s1.pdf]

Appendix

Items that assess different clusters of determinants

	Question or statement	Response
**Job characteristics**		
Managerial position	Are you currently in a managerial position?	Yes or no
Permanent contract	Do you currently have a tenured position?	Yes or no
**Perceiving the work environment as LG friendly**		
Having LG colleagues	Do you know any LGs at work who are open about their sexual orientation?	Yes or no
LG permissive atmosphere	The atmosphere at work is permissive of LGs (with a permissive atmosphere we mean that all employees feel respected and valued no matter their different characteristics)	1 = strongly disagree and 5 = strongly agree
Having witnessed homonegative reactions		
	Did you ever, during your work at the Flemish government, witness verbal intimidation of an LG colleague?	Yes or no
	Did you ever, during your work at the Flemish government, witness physical aggression towards an LG colleague?	Yes or no
	Did you ever, during your work at the Flemish government, witness an LG colleague being ignored because of his/her sexual orientation?	Yes or no
	Have you ever, during your work at the Flemish government, been confronted with the use of sexual innuendo towards LGs at work?	Yes or no
	Did you ever, during your work at the Flemish government, hear jokes about LGs in the workplace?	Yes or no
	Did you ever witness the use of abusive language (such as fagot, sissy, or dyke) by	Yes or no

	colleagues who were talking about LGs?	
	Did you ever, during your work at the Flemish government, hear gossip about LGs at work?	Yes or no
**Homonegative experiences**		
	Did you ever, during your work at the Flemish government, experience verbal harassment?	Yes or no
	Did you ever, during your work at the Flemish government, experience physical aggression?	Yes or no
	Did you ever, during your work at the Flemish government, experience sexual innuendo?	Yes or no
	Were you ever, during your work at the Flemish government, ignored by a colleague or superior because of your sexual orientation?	Yes or no
	Were you ever, during your work at the Flemish government, called names (such as fagot, sissy, dyke) by colleagues?	Yes or no
